# Histamine and Th2 cytokines independently and synergistically upregulate MMP12 expression in human M2 macrophages

**DOI:** 10.3389/fimmu.2024.1429009

**Published:** 2024-10-21

**Authors:** Alice Pereira da Fonseca, Stephan Traidl, Ralf Gutzmer, Katrin Schaper-Gerhardt, Thomas Werfel, Susanne Mommert

**Affiliations:** ^1^ Department of Dermatology and Allergy, Hannover Medical School, Hannover, Germany; ^2^ Department of Dermatology, Johannes Wesling Medical Center, Ruhr University Bochum, Minden, Germany

**Keywords:** atopic dermatitis, macrophages, MMP12, histamine, Th2 cytokines, dupilumab

## Abstract

Beyond Th2 cells and various immune cells, M2 macrophages have been identified in lesional skin of atopic dermatitis (AD) indicating their involvement in the disease’s underlying mechanisms. MMP12, a matrix-degrading enzyme, which is predominantly produced by macrophages, is increased in skin lesions of AD patients. In this study we investigated the expression of MMP12 mRNA in lesional AD skin at single cell level through RNA sequencing (scRNA-seq) and the expression of MMP12 in M2 macrophages from healthy individuals and AD patients in response to Th2 cytokines and histamine using quantitative PCR and ELISA. Additionally, we analyzed macrophages from dupilumab-treated AD patients using the same methods to assess the influence of Th2 cytokines on MMP12 expression *ex-vivo*. ScRNA-seq identified macrophages as the primary producers of MMP12 in lesional AD skin. *In-vitro*, both MMP12 mRNA and protein expression were significantly increased in monocytes during differentiation to M2 macrophages in the presence of histamine, of Th2 cytokines or of Th2 cytokines in combination with histamine. In M2 macrophages obtained from dupilumab-treated AD patients, the upregulation of MMP12 expression by IL-4 and IL-13 was attenuated. Our findings unveil a novel mechanism whereby Th2 cytokines and histamine regulate MMP12 expression, potentially impacting skin barrier homeostasis in AD.

## Introduction

Atopic dermatitis (AD) is an inflammatory skin disease with a chronic recurrent course. About 20% of the children and 3% of the adult population suffer from AD (1), making it one of the most common chronic inflammatory skin diseases (2).

One of the main functions of the skin is protection and defense against external influences. A reduced skin barrier is a feature of AD which increases the sensitivity to external exposures such as allergens or pathogens (3).

The mechanisms which contribute to an impaired skin barrier in AD are currently under ongoing research. MMP12, a matrix degrading enzyme, can degrade several substrates from the extracellular matrix, such as collagen IV, gelatin, fibronectin, laminin, vitronectin, elastin, fibrillin, α1-antitrypsin, and apolipoprotein A (4). Accordingly, MMP12 contributes to inflammatory tissue destruction (4).

Immunological processes involved in the pathomechanism of AD include the activation of different types of immune cells. In addition to T cells, also monocytes migrate into affected skin providing a niche for the differentiation to macrophages.

M1 and M2 macrophages represent two distinct functional states of macrophages, each characterized by specific surface markers, activation stimuli, secreted cytokines, and functions. M1 macrophages are typically identified by surface markers such as CD80, CD86, MHC class II, and the intracellularly expressed differentiation marker CD68 (5). M2 macrophages also express CD68, but also other molecules as M1 macrophages such as the scavenger receptor CD163, or the mannose receptor CD206 (6). M1 macrophages are primarily activated by Th1 cytokines, including IFN-γ and TNF-α, or by recognition of microbial products like bacterial lipopolysaccharides (LPS) (7). Upon activation, M1 macrophages secrete pro-inflammatory cytokines such as TNF-α, IL-1α, IL-1β, IL-6, IL-12, IL-23 and cyclooxygenase-2 (COX-2), along with reactive oxygen species (ROS) (8). M2 macrophages are activated by Th2 cytokines such as IL-4, IL-10, and IL-13 (9). M2 macrophages secrete key chemokines, including CCL13, CCL17 and CCL18 (6). Functionally, M1 macrophages play a crucial role in host defense by killing pathogens and displaying strong anti-tumoral activity. They promote inflammation, which can contribute to tissue damage if not properly regulated (8). In contrast, M2 macrophages play a role in tissue repair, angiogenesis, and metabolism (5).

In lesional AD skin, macrophages, and in particular M2 macrophages, are increased mainly in the upper dermis (10). Zhang et al. identified a population of macrophages expressing high mRNA levels of key molecules of M1 macrophages (TNF, IL1B, and IL18), a cluster expressing M2 macrophages related genes (CD163, CCL13, CCL17, and CCL18), and two closely related clusters of macrophages characterized by high expression of *IFITM3* and varying expression of IL1B in skin biopsies from AD patients by scRNA-seq (6).

The presence of IL-4 and IL-13 in lesional AD skin may increase the polarization of monocyte derived macrophages into M2 macrophages (11).

Histamine concentrations are elevated in inflamed skin and play an important role in the pathogenesis of AD by binding and signaling through four different histamine receptors (H1R – H4R) (12). Human M2 macrophages express H1R, H2R and H4R, but not H3R (13).

In epidermal and dermal samples of lesional and non-lesional skin MMP12 was detected under the top 25 upregulated genes in lesional skin in AD (14).

In a study investigating key genes and pathways in AD by bioinformatic analysis from the Gene Expression Omnibus (GEO) database, MMP12 was among the most highly upregulated genes in lesional skin biopsy samples when compared to samples from non-lesional skin (15).

Here we analyzed the expression of MMP12 in lesional AD skin, and investigated the regulation of MMP12 expression in *in-vitro* differentiated human M2 macrophages in response to Th2 cytokines and histamine, mediators which predominate the inflammatory milieu in the onset of AD (2).

## Methods

### Single cell RNA sequencing

Data from a previous study encompassing single cell RNA sequencing involved samples from 10 AD patients (6). In short, skin punch biopsies underwent dissolution with the Miltenyi Biotech skin dissociation kit for subsequent cell isolation, CD2 enrichment, pooling and loading into the chromium chip (10x Genomics, Pleasanton, CA, USA).

The CellRanger pipeline version 3.1.0 aligned reads to the human reference genome GRCH38. The resulting expression matrix was loaded into the Seurat package version 4.2.3 for downstream analyses. UMAP, feature plot and dot plot were created with the according functions. Differential expressed genes were analyzing using FindMarkers function. Only genes with log-fold change > 0.25 and expressed in at least 10% of cells were selected. Volcano plot visualization was performed using the EnhancedVolcano package.

### Differentiation of M2 macrophages

Residual blood samples (buffy coats) from platelet (PLT) apheresis disposables used for routine PLT collection and of regular anonymous healthy volunteers served as source material for the isolation of human peripheral blood mononuclear cells (PBMCs). Venous blood samples were taken for the isolation of PBMCs from untreated patients with AD (White, n = 12, 7 females and 5 males, mean age 35,4 years) and from AD patients who were treated with dupilumab (White, n = 15, 8 female and 7 males, mean age 38 years). The patients were treated with dupilumab for a minimum of 2 weeks and an average of 14.5 months. Patients fulfilled the requirements of the indication of system therapy with dupilumab according to current European guideline (16). AD patients were recruited from the Department of Dermatology and Allergy, Hannover Medical School, Hannover, Germany. AD was diagnosed according to the criteria of Hanifin and Rajka (17).

PBMCs from the different groups were separated by density gradient centrifugation on lymphoprep (Fresenius Kabi Norge AS, Oslo, Norway). With a seeding density of 1x10^6^ cells pro well, PBMCs were plated in a 24 well plate in Iscoves Medium supplemented with AB serum (2,5% v/v).

We differentiated PBMCs/monocytes with M-CSF into M2 macrophages comparable to our previous studies (11) according to the protocol of Promocell (18). To attach the monocytes, cells were incubated for 1.5 h at 5% CO2 and 37°C on plastic dishes. Non-adherent cells were removed by vigorously washing of adherent cells three times with PBS. An appropriate amount of RPMI 1640, supplemented with 2 mM l-glutamine, 100 mg/ml penicillin/streptomycin, 12 mM Hepes, and 5% v/v human AB serum heat inactivated (PAN-Biotech, Aidenbach, Germany; all other media components from Biochrom, Berlin, Germany) and 10 ng/ml macrophage colony-stimulating factor (M-CSF) (R&D, San Diego, CA, USA) was added. Cells were incubated at 37°C and 5% CO_2_.

At day 5, 300 µl fresh medium was added. At day 8 the medium was completely removed, fresh medium was added. The differentiation process was controlled at day 8 and 10: M2 macrophages appeared as adherent cells showing a typical morphology with a prominent nucleus, scattered cytoplasm, and a couple of pseudopodia. The phenotype of the cells was previously published by us (13).

### Stimulation of monocytes during their differentiation to M2 macrophages

For assessment of MMP12 mRNA expression and protein concentration during the differentiation process of the cells, monocytes were stimulated during their differentiation to M2 macrophages. The stimuli were added to the media from day 1 until the analysis of MMP12 expression on day 10, with fresh additions made at each media change.

The following reagents were used: Histamine (Alk-Scherax, Wedel, Germany), 2-pyridylethylamine (Tocris Bioscience, Bristol, UK) as selective H1R agonist, amthamine (Tocris Bioscience) as selective H2R agonist, ST-1006 (Institute of Pharmaceutical and Medicinal Chemistry, Heinrich Heine University, Düsseldorf, Germany) as a H4R agonist (19) or with IL-4 (20 ng/ml) (R&D Systems) or IL-13 (15 ng/ml) (R&D Systems). All histamine receptor ligands were used at a concentration of 10 µM.

In extensive previous studies, we have shown that 10 μM is the optimal concentration to demonstrate and reproduce robust histamine receptor agonist mediated effects (20).

### Stimulation of fully differentiated M2 macrophages

For assessment of MMP12 mRNA expression and protein concentration in fully differentiated M2 macrophages, cells were isolated from PBMCs from anonymous healthy donors and from AD patients treated with or without dupilumab. M2 macrophages from anonymous healthy donors were stimulated with histamine (10 µM), with IL-4 (20 ng/ml) (R&D Systems) or IL-13 (15 ng/ml) (R&D Systems) for 6 h or 24 h or left non-stimulated.

M2 macrophages obtained from the two AD patient groups were stimulated at day 8 with IL-4 (20 ng/ml) (R&D Systems) or IL-13 (15 ng/ml) (R&D Systems) for 48 h or left non-stimulated.

### Reagents to block the IL-4R type I and IL-4/IL-13R type II receptor complex subunits, the Janus/Tyrosine kinases and the transcription factor activator protein-1

To block the IL-4R type I and IL-4/IL-13R type II receptor complex subunits, the Janus/Tyrosine kinases (JAK/TYK) and the transcription factor activator protein-1 (AP-1), the following reagents were used in the culture systems: i) Antibodies against the IL-4R type I and IL-4/IL-13R type II receptor complex subunits: Monoclonal mouse IgG4 antibody dupilumab (Dupixent, Sanofi-Aventis GmbH, Frankfurt am Main, Germany) inhibiting the IL-4Rα chain; polyclonal goat IgG antibody (common γ chain/IL-2Rγ R&D Systems) inhibiting the IL-4R common γ chain/IL-2Rγ; monoclonal mouse antibody (Aldevron, Freiburg, Germany) inhibiting the IL-13R subunit α1. ii) Inhibitors of the IL-4R type I and IL-4/IL-13R type II receptor down-stream signaling adaptor molecules: T-5224 (10 µM) (Chayman, Chemical, USA) for activator protein-1 (AP-1); PF 06651600 malonate (10 µM) (Tocris Bioscience, Bristol, UK) as a potent and selective Janus kinase 3 (JAK3) inhibitor; TC JL 37 (10 µM) (Tocris Bioscience, Bristol, UK) as a potent inhibitor of Tyrosine kinase 2 (TYK2).

The blocking reagents were added to the fully differentiated M2 macrophages isolated from PBMCs from anonymous healthy donors 20 minutes before stimulation with IL-4 (20 ng/ml) (R&D Systems) or IL-13 (15 ng/ml) (R&D Systems) for 48 h.

The cells we used for this experiment were previously published for other targets in Mommert et al. (21).

### Quantitative PCR

Total RNA was isolated using the RNeasy kit (Qiagen, Hilden, Germany) according to the manufacturer’s instructions. The cDNA was synthesized by reverse transcription (QuantiTect reverse transcription kit, Qiagen, Hilden, Germany). Quantitative PCR (q-PCR) was performed in real time with Quantitect^®^ primer assays for MMP12 (QT01004472) and RPS 20 (ribosomal protein S20) (QT00079247) using SYBR^®^ Green according to the manufacturer’s instructions (Qiagen, Hilden, Germany) using the LightCycler 1.5 and 480. The amount of the target mRNA relative to the amount of the reference gene, ribosomal protein S20 (rps 20), mRNA in the same sample was calculated using the comparative Ct method also known as the [delta] Ct method and target/reference ratios are depicted. Otherwise Ct values of the targets are normalized to the Ct values of the housekeeping gene ribosomal protein S20 (rps 20) and expressed as normalized mRNA ratio calculated by the [delta] [delta] Ct method to the non-stimulated or to the IL-4 or IL-13 stimulated samples.

### ELISA

*In-vitro* differentiated M2 macrophages isolated from PBMCs from anonymous healthy donors were incubated at day 8 with IL-4 or IL-13 for 48 h. The concentrations of MMP12 were analyzed in cell-free supernatants with ELISA according to the manufacturer’s instructions (Abcam, Cambridge, England).

### Statistics

For statistical analyses, the software GraphPad Prism Version 8.0 was used (GraphPad software, San Diego, CA, USA). First, we performed methods to test the normal Gaussian distribution of the data. In all our experiments due to the individual variations of the data the normality tests failed. The non-parametric tests Wilcoxon matched-pairs signed rank test or Friedman Dunn’s Multiple Comparison test selected pairs was performed and the median is shown in the graph. Unpaired samples from two groups were evaluated using Mann–Whitney test. For all calculations, a 95% confidence range was defined. A p-value < 0.05 was regarded as statistically significant (p < 0.05 was labelled with *, p < 0.01 was labelled with **, p < 0.001 was labelled with ***, p < 0.0001 was labelled with ****).

## Results

### Single cell RNA sequencing of AD skin reveals high expression of MMP12 in macrophages when compared to other immune cells

**Figure 1:**
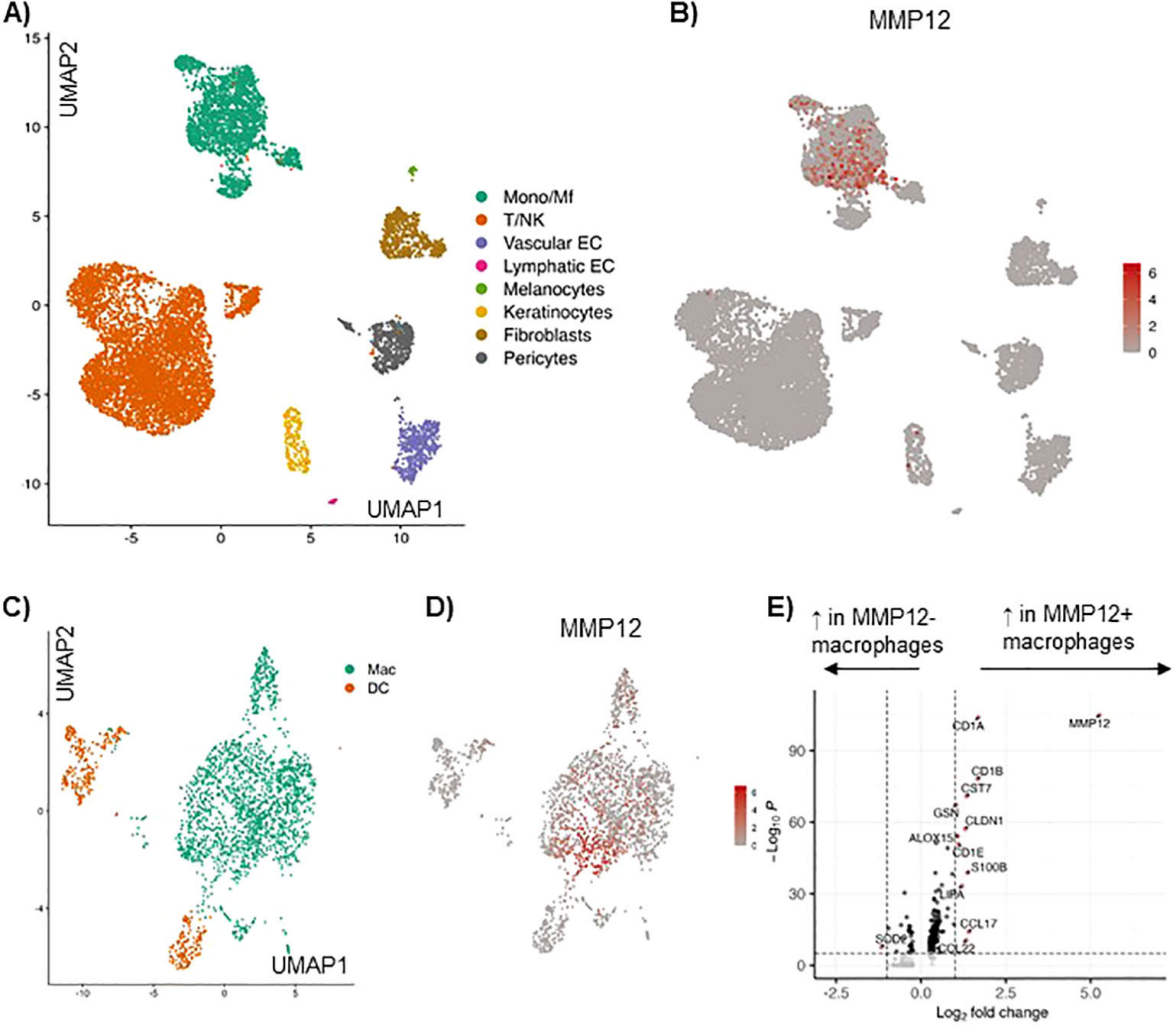
Single cell RNA sequencing of AD skin reveals high expression of MMP12 in the myeloid cell cluster, in particular in macrophages and monocytes, as compared to other immune cells. **(A)** UMAP showing identified cell clusters in lesional skin in AD, cumulative data from 10 skin biopsies, for details see Zhang et al. (6). **(B)** Feature plot displaying the MMP12 expression in each cell cluster. **(C)** Identification of macrophages (Mac) and dendritic cells (DC) in the myeloid cell cluster in lesional AD skin by calculating their marker genes. **(D)** MMP12 expression displayed by a feature plot. **(E)** Differentially expressed genes in MMP12 negative and positive macrophages (volcano plot).

Analysis of single cell RNA sequencing of AD skin based on our previously published data set (6), identified the major cell types by calculating marker genes for each cell subcluster (**Figure 1A**). The expression of MMP12 was analyzed for each cell subtype. In the myeloid cell cluster high expression of MMP12 was detected (**Figure 1B**). Resolution of the myeloid cell cluster by subclustering led to classification of a population of macrophages and different populations of dendritic cells (**Figure 1C**).

In this cell population macrophages showed the highest MMP12 mRNA expression (**Figure 1D**). Many genes were differentially expressed in MMP12 negative compared to MMP12 positive macrophages. MMP12 clustered with a high expression of CCL17, a M2 macrophage specific chemokine and well established biomarker for AD (**Figure 1E**).

### Histamine, IL-4, IL-13 and a combination either IL-4 or IL-13 together with histamine upregulate MMP12 mRNA expression in M-CSF differentiated macrophages

**Figure 2:**
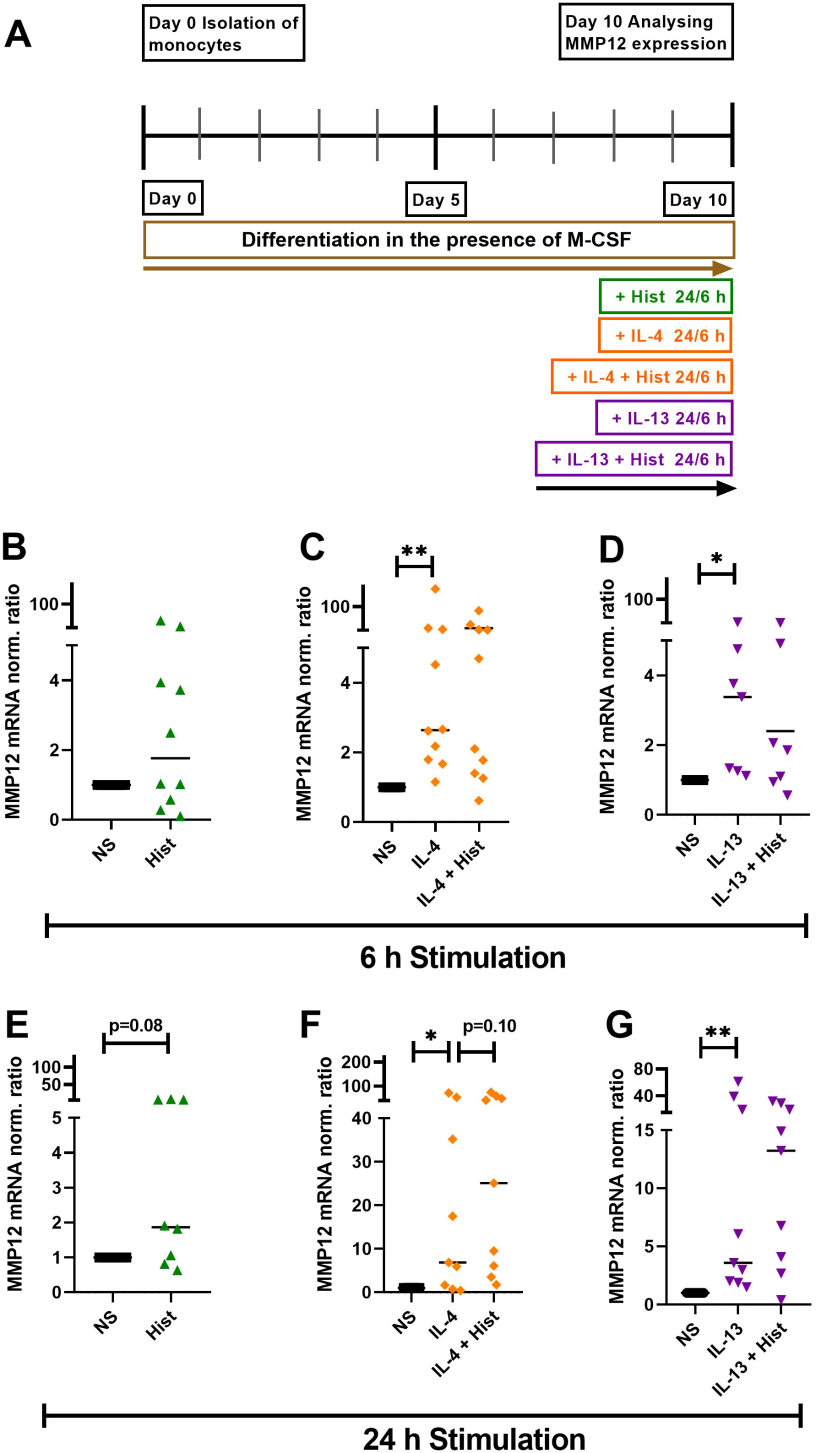
Histamine, IL-4, IL-13 and either IL-4 or IL-13 together with histamine upregulate MMP12 mRNA expression in M-CSF differentiated macrophages. **(A)** Human monocytes were isolated from PBMCs obtained from anonymous healthy donors and differentiated into M2 macrophages in the presence of M-CSF (10 ng/ml) over 10 days. M2 macrophages were stimulated for 6 h and 24 h as indicated or left non-stimulated. MMP12 mRNA expression in response to histamine (Hist, 10 µM), **(B)** after 6 h, **(E)** after 24 h; in response to IL-4 (20 ng/ml) or IL-4 + histamine **(C)** after 6 h, **(F)** after 24 h; in response to IL-13 (15 ng/ml) or IL-13 + histamine, **(D)** after 6 h, **(G)** after 24 h. MMP12 mRNA expression levels were quantified by qPCR and calculated by the [delta] [delta] Ct method and normalized to the non-stimulated samples. Data are shown as individual values; horizontal bars indicate the medians. Significant differences, as determined by the Wilcoxon matched-pairs signed-rank test are indicated as follows: *p < 0.05; **p < 0.01; (**B, C**, n = 10 independent donors and experiments); (**D**, n = 7 independent donors and experiments); (**E,** n = 8 independent donors and experiments); (**F, G**, n = 9 independent donors and experiments), NS, non-stimulated.

We stimulated M-CSF differentiated macrophages with histamine, IL-4, IL-13 or a combination either IL-4 or IL-13 together with histamine for 6 h and 24 h, while maintaining a non-stimulated control group (**Figure 2A**). We observed a trend of increased MMP12 mRNA expression induced by histamine after 6 h, which became more pronounced after 24 h (**Figures 2B, E**). Both IL-4 (**Figures 2C, F**) and IL-13 (**Figures 2D, G**) significantly upregulated MMP12 mRNA expression after 6 h continued after 24 h. The combination of histamine with either IL-4 (**Figures 2C, F**) or IL-13 (**Figures 2D, G**) showed a trend towards upregulation of MMP12 mRNA expression only after 24 h.

### Histamine upregulates the expression of MMP12 during differentiation from monocytes to M2 macrophages and in IL-4- and IL-13-activated macrophages

**Figure 3:**
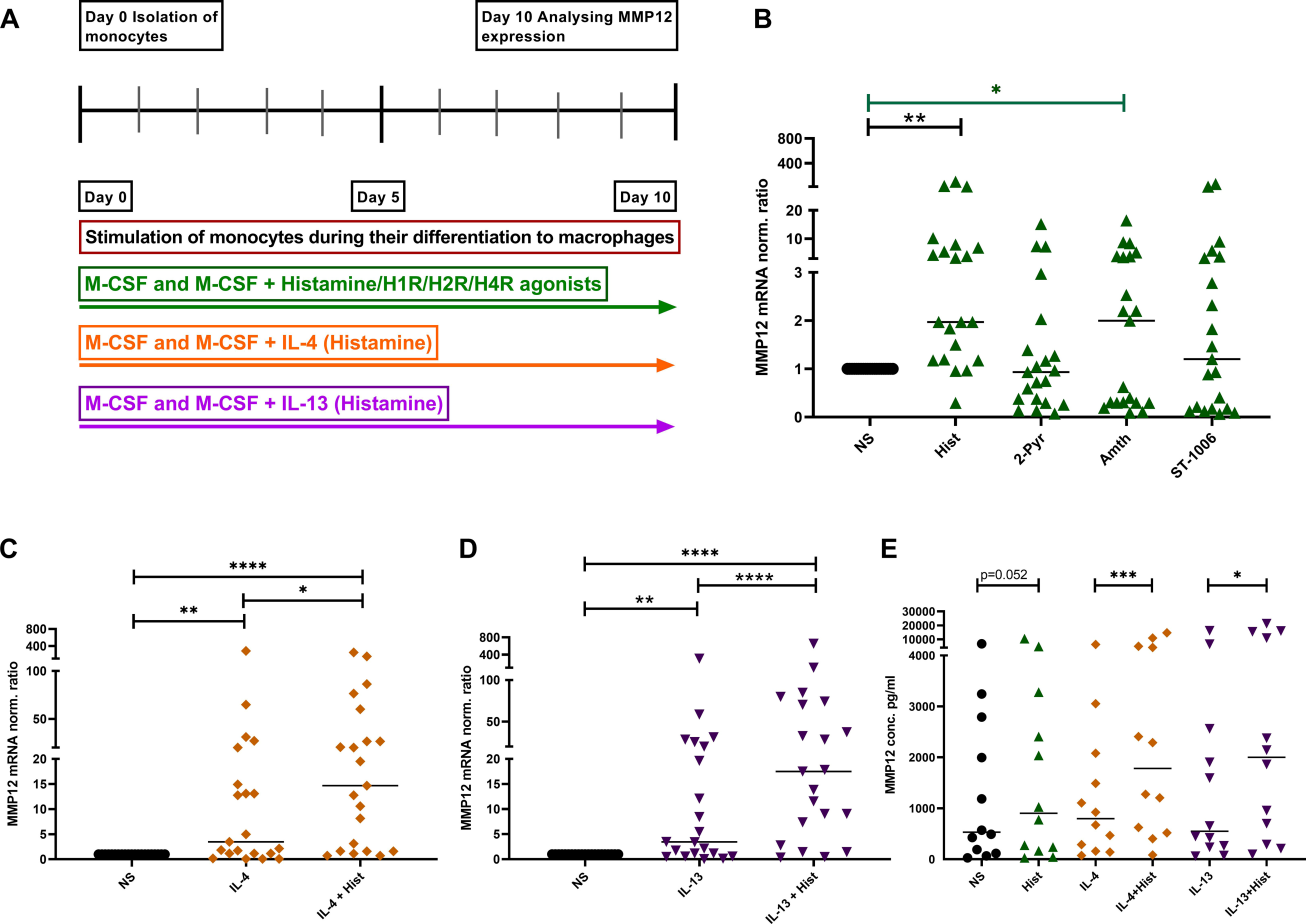
Upregulation of MMP12 mRNA expression and secretion by histamine during the differentiation of human monocytes into M2 macrophages and in IL-4- and IL-13-activated M2 macrophages. **(A)** Human monocytes were isolated from PBMCs obtained from anonymous healthy donors and differentiated into M2 macrophages in the presence of M-CSF (10 ng/ml) and different stimuli as indicated over 10 days. **(B)** MMP12 mRNA expression at day 10 after stimulation with histamine (Hist, 10 µM), 2 –pyridylethylamine (2-Pyr, H1R agonist, 10 µM), amthamine (Amth, H2R agonist, 10 µM) or ST-1006 (H4R agonist, 10 µM). **(C)** with IL-4 (20 ng/ml) or IL-4 + histamine. **(D)** MMP12 mRNA expression at day 10 after stimulation with IL-13 (15 ng/ml) or IL-13 + histamine. MMP12 mRNA expression levels were quantified by qPCR and calculated by the [delta] [delta] Ct method and normalized to the non-stimulated samples. **(E)** Assessment of MMP12 secretion by ELISA. Data are shown as individual values; horizontal bars indicate the medians. Significant differences, as determined by the Friedman Dunn’s multiple comparison test in **(B)** (bars and stars in black) or by the Wilcoxon matched-pairs signed-rank test in **(B)** (bars and stars in green) and in **(C–E)** are indicated as follows: *p < 0.05; **p < 0.01; ***p < 0.001; ****p < 0.0001; (**B–D**, n = 21 independent donors and experiments); (**E**, n = 12 independent donors and experiments); NS, non-stimulated.

We stimulated monocytes obtained from anonymous healthy donors during their differentiation to macrophages with histamine or with the different histamine receptor agonists, with IL-4, IL-13 or a combination of histamine with either IL-4 or IL-13 (**Figure 3A**). Histamine and the selective H2R agonist amthamine led to a significant upregulation of MMP12 mRNA in *in-vitro* differentiated M2 macrophages. Moreover, the presence of the selective H4R agonist ST-1006 led to an upregulation of MMP12 mRNA expression by trend (**Figure 3B**).

To demonstrate that histamine or amthamine treatment during macrophage differentiation specifically enhances MMP12 mRNA expression, independent of their potential role in promoting M2 polarization, we conducted an experiment where macrophages were treated with histamine and the H2R agonist amthamine while a separate group of cells remained untreated throughout differentiation. After 10 days, we analyzed the mRNA expression levels of MMP12, the macrophage differentiation marker CD68, the scavenger receptor CD163, and the M2 macrophage-specific chemokines CCL17 and CCL18 in parallel in cells from the same donors.

Again, we observed a significant upregulation of MMP12 mRNA expression following treatment with histamine and amthamine during macrophage differentiation. A moderate upregulation of CCL18 mRNA expression was also detectable. In contrast, the mRNA expression levels of the macrophage differentiation marker CD68, the scavenger receptor CD163, and the M2 macrophage-specific chemokine CCL17 remained unchanged compared to untreated cells (**Supplementary Figure 1**).

These findings clearly indicated that neither histamine nor amthamine influences macrophage differentiation or upregulates the associated differentiation markers.

The presence of IL-4 or IL-13 during the differentiation induced a moderate, but significant upregulation of MMP12 expression at mRNA and protein level which was significantly increased by histamine (**Figures 3C–E**). In addition, we investigated the immune response of M2 macrophages from patients with AD and healthy donors, focusing on the expression of MMP12 after stimulation with IL-4 and IL-13 for 48 h.

We detected that M2 macrophages from AD patients showed higher expression of MMP12 at baseline and a stronger upregulation of MMP12 in response to IL-4 and IL-13 compared to cells from healthy anonymous donors (**Supplementary Figure 2**).

### The upregulation of MMP12 mRNA expression and secretion in M2 macrophages activated by IL-4 or IL-13 is attenuated in cells from AD patients treated with dupilumab

**Figure 4:**
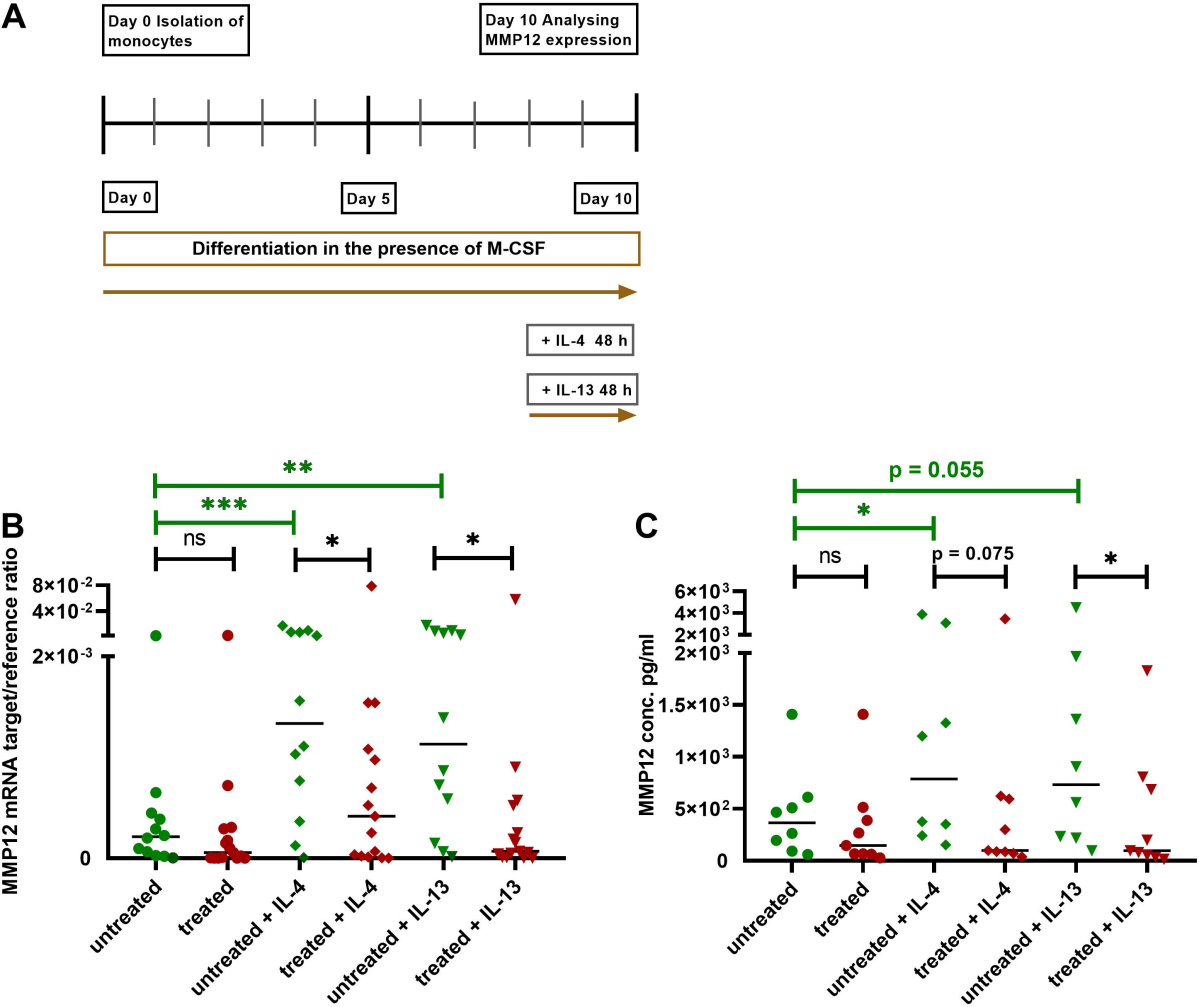
Attenuation of upregulation of MMP12 mRNA expression and secretion in IL-4- or IL-13-activated M2 macrophages from AD patients treated with dupilumab. **(A)** Human monocytes were isolated from PBMCs from AD patients treated with dupilumab over the period of at least 2 weeks and an average of 14,5 months. Treated means: AD patients were treated *in-vivo* with dupilumab before cells were differentiated into M2 macrophages *in-vitro*. Cells from untreated AD patients served as controls. Monocytes were differentiated over the period of 8 days into M2 macrophages in the presence of M-CSF (10 ng/ml). At day 8 the cells were activated with IL-4 (20 ng/ml) or IL-13 (15 ng/ml) for 48 h. **(B)** Target/reference ratios of MMP12 mRNA/rps 20 mRNA expression were analyzed by qPCR and calculated by the [delta] Ct method. **(C)** MMP12 secretion was assessed by ELISA. Data are shown as individual values with medians. Significant differences, as determined by the Mann-Whitney test (bars and stars in black) for IL-4- and IL-13-activated cells from dupilumab treated AD patients compared to cells from untreated AD patients or by the Wilcoxon matched-pairs signed-rank test (bars and stars in green) for non-activated cells and activated cells with IL-4 or IL-13 in the same patient group in **(B, C)** are indicated as follows: *p < 0.05; **p < 0.01; ***p < 0.001; (**B**, n = 12 (untreated) and 15 (treated) independent donors and experiments); (**C**, n = 8 (untreated) and 9 (treated) independent donors and experiments); ns, not significant.

We isolated monocytes from AD patients treated with dupilumab as well as from untreated AD patients. After differentiation of monocytes to M2 macrophages in the presence of M-CSF for 8 days, cells from both groups were activated with IL-4 or IL-13 for 48 h and MMP12 expression was measured at mRNA and protein level (**Figure 4A**). Most importantly, the IL-4- and IL-13-induced upregulation of MMP12 at mRNA and protein level was significantly attenuated in M2 macrophages from AD patients treated with dupilumab when compared to cells from untreated patients (**Figures 4B, C**).

### Blocking the IL-4Rα and IL-13Rα1 subunits of the IL-4R type I and IL-4/IL-13R type II *in-vitro* inhibits the IL-4- or IL-13-induced upregulation of MMP12 mRNA expression in M2 macrophages

To elucidate the role of the IL-4R type I and IL-4/IL-13R type II receptor subunits in detail and to confirm the effect of dupilumab in a controlled *in-vitro* approach on MMP12 production, we performed the following experiments with monocytes from anonymous healthy donors. The signaling pathways of the IL-4 type I and IL-4/IL-13 type II receptors regulating the histamine receptor expression levels in eosinophils or M2 macrophages were investigated in previous studies using blocking antibodies against the receptor subunits and inhibitors of the associated Janus/Tyrosine kinases (21, 22). Both studies evaluated antibodies and inhibitors in preliminary experiments and showed that different blocking antibodies against receptor subunits had distinct effects on receptor regulation as well the use of Janus/Tyrosine kinase inhibitors produced specific effects on signaling pathways associated with IL-4 and IL-13 receptors. This specificity provides evidence that the observed effects were due to targeted blocking rather than non-specific interactions.

**Figure 5:**
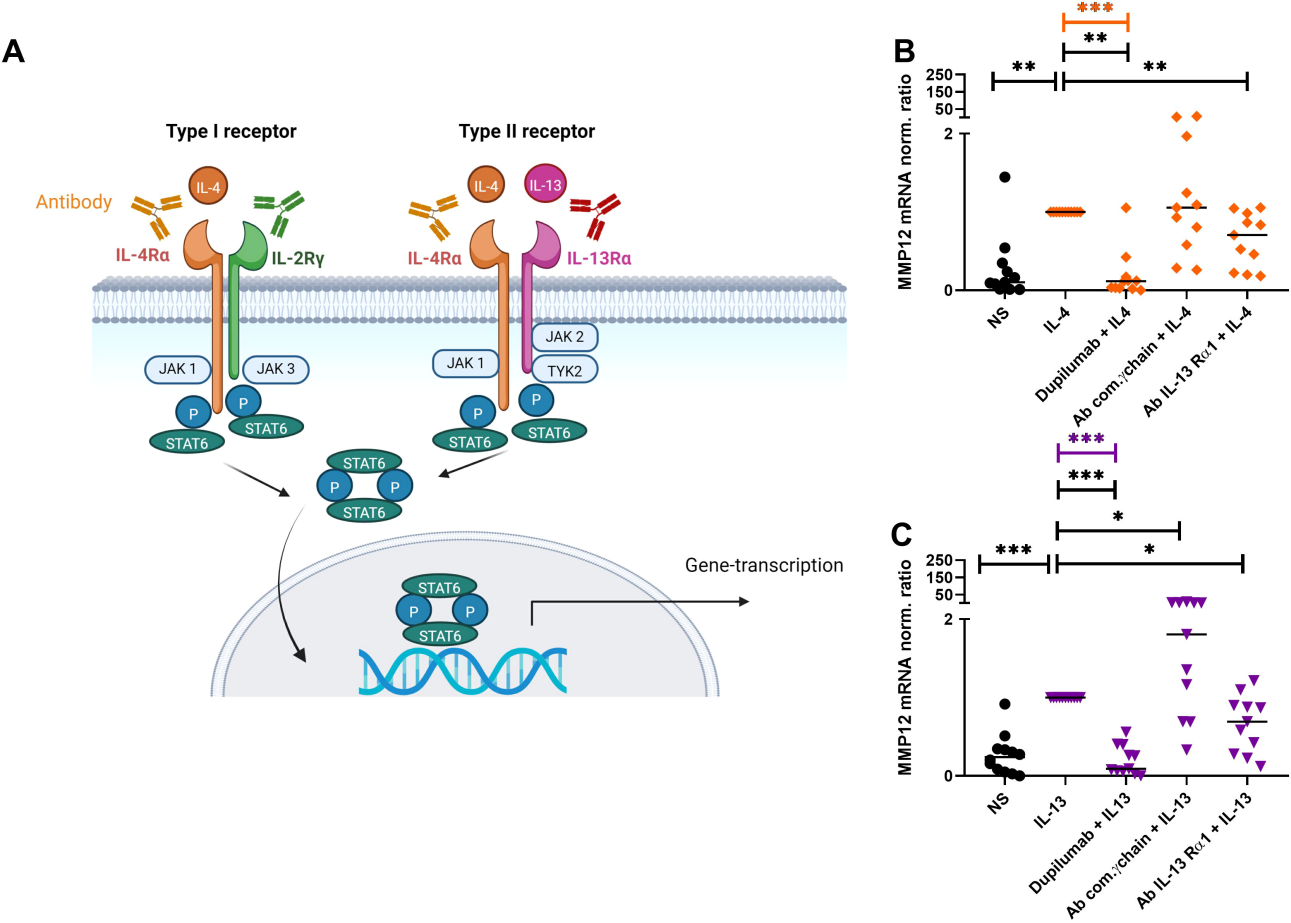
Attenuation of IL-4- or IL-13-induced upregulation of MMP12 mRNA expression in M2 macrophages by targeting IL-4R type I and IL-4/IL-13R type II with anti IL-4Rα or anti IL-13Rα1 antibodies. **(A)** Type I (IL-4) receptor, consisting of the IL-4 receptor alpha (IL-4Rα) and the common γ-chain subunits (IL-2Rγ), type II (IL-4/IL-13) receptor, consisting of the IL-4Rα and IL-13Rα1 subunits, trigger activation of signaling adaptor molecules that induce recruitment and phosphorylation of Signal Transducer and Activator of Transcription 6 (STAT6) to activate specific gene transcription programs. Schematic overview created with BioRender.com. **(B, C)** After differentiation in the presence of M-CSF M2 macrophages were pre-incubated for 20 min with antibodies [dupilumab (10 µM), a monoclonal antibody which blocks the IL-4Rα subunit a polyclonal goat IgG antibody against the common γ chain/IL-2Rγ (20 µM) a monoclonal antibody (10 μM) against the IL-13Rα1 subunit] targeting the extracellular domains of IL-4R type I or IL-4/IL-13R type II receptor complex subunits at day 8. After 20 minutes M2 macrophages were stimulated with IL-4 (20 ng/ml) or IL-13 (15 ng/ml) for 48 h. **(B)** MMP12 mRNA expression of IL-4-activated M2 macrophages, **(C)** MMP12 mRNA expression of IL-13-activated M2 macrophages.Target/reference ratios of MMP12 mRNA/rps 20 mRNA expression were analyzed by qPCR and normalized to the IL-4 or IL-13 stimulated samples calculated by the [delta] [delta] Ct method and expressed as normalized ratio. Data are shown as individual values with medians. Significant differences, as determined by the Friedman Dunn’s multiple comparison test (bars and stars in orange/pink) or by the Wilcoxon matched-pairs signed-rank test (bars and stars in black) are indicated as follows: *p < 0.05; **p < 0.01; ***p < 0.001. **(A, B)** (n = 11 independent donors and experiments), NS, non-stimulated.

After differentiation into M2 macrophages, the cells were treated with antibodies against the subunits of IL-4R type I (IL-4Rα; dupilumab) or the common γ (gamma) chain (IL-2Rγ) or IL-4/IL-13R type II subunits (IL-4Rα (dupilumab) or IL-13Rα1) for 20 minutes before stimulation with IL-4 or IL-13 for 48 h (**Figure 5A**). We observed an attenuation of the IL-4- or IL-13-induced upregulation of MMP12 expression by blocking the IL-4Rα or the IL-13Rα1 subunits but not by blocking the IL-2Rγ chain of the IL-4R Type I receptor (**Figures 5B, C**). In contrast, we observed an upregulation of MMP12 expression using the polyclonal goat IgG antibody against the common IL-2Rγ chain for blocking the IL-4 type I receptor. Macrophages beyond the IL-4R also express the receptor for IL-2 containing the common γ chain. We assume that the polyclonal goat IgG antibody may have off target effects instead of blocking it activates IL-4 and IL-2 receptor signaling and macrophage activation in part, resulting in upregulation of MMP12 expression.

### Blocking IL-4R type I and IL-4/IL-13R type II signaling adaptor molecules by selective inhibitors prevents the IL-4- or IL-13-induced upregulation of MMP12 mRNA expression in M2 macrophages mainly via type II receptor

**Figure 6:**
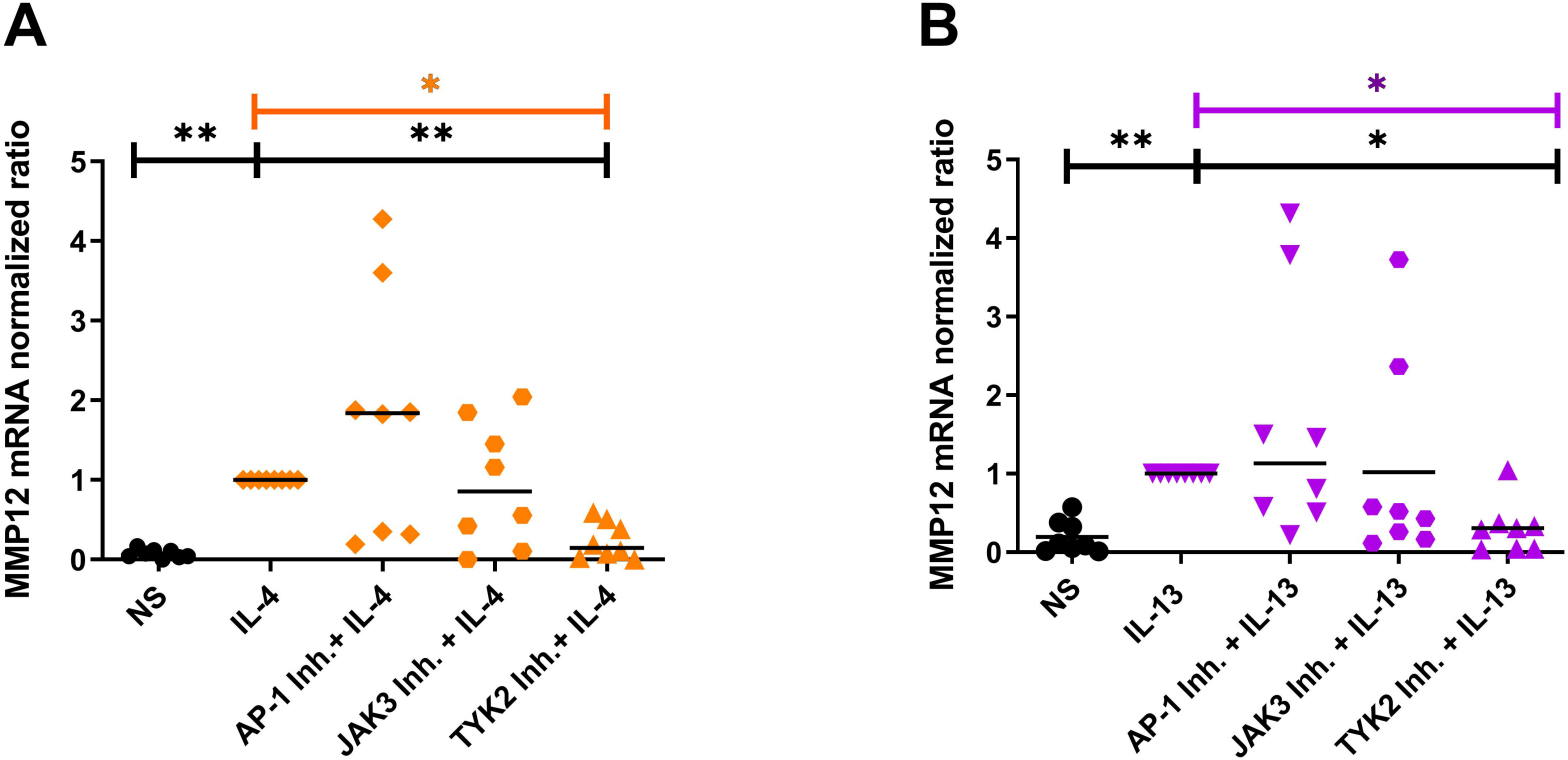
Blocking the IL-4R type I and IL-4/IL-13R type II signaling adaptor molecules by selective inhibitors prevents the IL-4- or IL-13-induced upregulation of MMP12 mRNA expression in M2 macrophages mainly via type II receptor. **(A, B)** In the presence of M-CSF differentiated macrophages from PBMCs obtained from anonymous healthy donors were stimulated at day 8 with IL-4 (20 ng/ml) or IL-13 (15 ng/ml) for 48 h. 20 minutes before stimulation with IL-4 or IL-13 the down-stream signaling adaptor molecules were blocked by selective inhibitors: AP-1 Inh. = T-5224 (10 µM) selective for activator protein-1; JAK3 Inh. = PF 06651600 malonate (10 µM) selective for JAK3; TYK2 Inh. = TC JL 37 (10 µM) selective for TYK2. **(A)** MMP12 mRNA expression in IL-4 stimulated M2 macrophages, **(B)** MMP12 mRNA expression in IL-13 stimulated M2 macrophages. Significant differences, as determined by Friedman Dunn’s multiple comparison test selected pairs (bars and stars in orange/pink) and as determined by the Wilcoxon matched-pairs signed rank test (bars and stars in black) are indicated as follows: *p < 0.05; **p < 0.01; medians are shown in the graphs. NS, non-stimulated; **(A, B)** (n = 8 independent donors).

In order to identify the receptor that is mainly responsible for the upregulation of MMP12 expression, we inhibited the receptor-associated signaling molecules with their selective inhibitors. Monocytes from anonymous healthy donors were differentiated to M2 macrophages, the cells were treated for 20 minutes with inhibitors of the down-stream regulators JAK3, TYK2 and of the activator protein-1 (AP-1) before IL-4, IL-13 cytokine stimulation for 48 h. We observed that only the inhibition of TYK2 was able to prevent the IL-4- and IL-13- induced up-regulation of MMP12 mRNA expression pointing to a prevailing role of the IL-4/IL-13R type II in this reaction (**Figures 6A, B**). AP-1 inhibitor treatment showed a tendency to increase MMP12 expression. The regulation of MMP12 is highly complex. AP-1, which is composed of the proteins c-fos and c-jun is known to activate MMP12 expression in response to certain stimuli but it also interacts with other regulatory elements that may influence MMP12 expression. This means that the inhibition of AP-1, cells may activate alternative signaling pathways or transcription factors that can compensate for the loss of AP-1 activity. It should be considered that AP-1 inhibition may generally suppress MMP12 expression, however the potential for increased MMP12 expression in macrophages following such treatment remains an open question and would benefit from further research to elucidate these dynamics (23).

## Discussion

MMP12, a matrix-degrading enzyme, and a potent inflammatory mediator, is elevated in lesional skin of AD patients compared to normal skin (24).

To uncover which cells are responsible for the elevated concentrations of MMP12 in AD, we analyzed single cell RNA seq data from AD skin from a study published by us in detail previously (6). This analysis revealed that macrophages are the key cells expressing MMP12 in the skin of AD. Our findings are in line with Suzuki et al. who showed in skin biopsies from mice that MMP12 is mainly produced by M2 macrophages (25).

MMP12 is associated with macrophage activity. MMP12 released from pro-inflammatory GM-CSF polarized human M1 macrophages is involved in vascular homeostasis (26). MMP12 produced by M2 macrophages plays a role in the development of contact hypersensitivity (27). However, the inconsistent association of MMP12 with the different macrophage subtypes and the varying expression levels of MMP12 depending from the tissue microenvironment and different stimuli makes MMP12 not suitable for macrophage subtype identification (27, 28).

To simulate the allergic Th2-polarized *in-vivo* environment more accurately, we stimulated M2 macrophages with histamine, IL-4, IL-13, or a combination of histamine with either IL-4 or IL-13. We applied these stimuli both for short-term exposure and throughout the entire macrophage differentiation process. The elevated MMP12 mRNA expression levels observed after short-term stimulations of 6 hours and 24 hours indicated the significant upregulation of MMP12 mRNA expression, which was detected when histamine IL-4, IL-13 and their combinations were added throughout the entire differentiation process of M2 macrophages. The interaction between differentiation and external signaling in modulating MMP12 levels suggests that MMP12 is not merely a byproduct of differentiation but is actively regulated during this process.

In addition previous data obtained by flow cytometry showed that the presence of histamine and various histamine receptor agonists affects the expression of the differentiation marker CD68 and the M2 macrophage marker CD163 in a partially significant but only very moderate and non-synergistic manner (29).

Our findings assessing the mRNA expression levels of MMP12 and various differentiation markers in parallel, clearly demonstrated a direct upregulation of MMP12 in response to histamine and amthamine, but there was no influence on macrophage differentiation or upregulation of the associated differentiation markers.

However, in general we observed individual differences and increased variability in MMP12 expression levels between the donors. This variability may be attributed to the unique characteristics of each donor such as demographic factors, genetic profiles, or medical history. The distinct expression levels of MMP12 in AD patients and healthy donors in our study reflect the impact of these individual donor characteristics on cellular function and response. This observation should be addressed in future studies in larger patient cohorts.

The crosstalk of histamine with IL-4 and IL-13, in particular the observation that Th2 cytokines act as priming factors for histamine mediated effects (30), may play a role in this scenario. Amplication of cellular functions by histamine after IL-4 priming has been described for other cells before: In human umbilical vein endothelial cells IL-4 primes the cells to respond to histamine with an increase of PGE2 release. In these cells IL-4 had no effects on signaling pathways but upregulated the H1R expression assumed to contribute to the enhanced responsiveness of histamine (30). In human M2 macrophages, Th2 cytokines were shown to upregulate H2R and H4R expression (21) which are involved in the effect of histamine increasing IL-4- or IL-13-induced CCL17 (11) and CCL18 expressions (9). We assume that the upregulation of the histamine receptors by IL-4 or IL-13 during the activation of macrophages (29) represents a critical event also for the effects of histamine enhancing the IL-4/IL-13-induced MMP12 production in M2 macrophages only in long-term stimulations during the differentiation process.

As previously reported, treatment of AD patients with dupilumab, a humanized monoclonal IgG4 antibody that binds to the IL-4Rα subunit of IL-4R type I and of IL-4/IL-13R type II, downregulated the expression of MMP12 in the skin of AD patients in a dose-dependent manner during treatment (31).

In support of the hypothesis that macrophages are one of the main producers of MMP12 in AD skin and to rule out the role of IL-4 and its cognate type I and type II receptors in MMP12 production, we isolated monocytes from AD patients treated with dupilumab as well as from untreated AD patients in the present study. We showed that the upregulation of MMP12 expression in IL-4- and IL-13-activated macrophages is markedly attenuated in cells from AD patients treated with dupilumab. These novel observations on macrophages which were cultivated up to 10 days after collection from the AD patients demonstrate that clinical treatment with dupilumab exerts a long lasting inhibitory activity on monocytes and macrophages under Th2 cytokine-induced *in-vitro* effects.

Our results could be explained by observations of Heeb and Boyman, who showed that treatment with dupilumab leads to occupation and downregulation of IL-4Rα surface expression on immune cells including monocytes. In their study, engagement of the IL-4Rα with dupilumab led to endocytosis of this complex. Of note, even after more than a year of therapy with dupilumab a “dormant” intracellular IL-4Rα pool which remained unoccupied could be detected, leading to a reservoir for immediate IL-4Rα upregulation (32).

Importantly, M2 macrophages from AD patients showed higher expression of MMP12 at baseline and a stronger upregulation of MMP12 in response to IL-4 and IL-13 compared to cells from healthy anonymous donors. AD is characterized by a dominant Th2 immune response, which is driven by cytokines such as IL-4 and IL-13. Macrophages from AD patients are supposed to be in a hyperactive state due to the chronic inflammatory milieu created by Th2 cytokines. We assume that the active state of the cells contributes to the increased production of MMP12 per se and in response to IL-4 and IL-13 in *in-vitro* experiments with M2 macrophages from AD patients compared to macrophages from healthy donors.

Selective blocking of the extracellular domain of the IL-4Rα and IL-13Rα1 subunits with antibodies resulted in the attenuation of IL-4- or IL-13-induced upregulation of MMP12 mRNA expression. MMP12 expression was evaluated only at the transcriptional level, since the direct measurement of MMP12 gene activation provides immediate insight into signal transduction from receptor manipulation to the target gene and allows detection of even small changes in MMP12 gene expression. To provide more insight in the complexity of IL-4/IL-13 signaling pathway further research with complementary methods such as protein analyses and functional studies is recommended.

In contrast, blocking the common IL-2Rγ chain of IL-4R type I had no effect, suggesting a dominant role of the IL-4/IL-13R type II receptor in this response. A similar result was detected in a previous work where blocking of the IL-4/IL-13R type II receptor led to an inhibition of Th2 cytokine-induced H4R upregulation (21).

Targeting the IL-4 receptor complexes I or II induce intracellular signaling. Binding of IL-4 to the type I receptor activates JAK1 and JAK3, whereas JAK1 and TYK2 are the tyrosine kinases involved in IL-4/IL-13 signaling via the type II receptor (33). To identify the receptor involved in the up-regulation of MMP12, we inhibited the down-stream regulators JAK3, TYK2 and the activator protein-1 and (AP-1) before stimulation M2 macrophages with IL-4 or IL-13. Interestingly, only the inhibition of TYK2 was able to prevent the IL-4- and IL-13 –induced up-regulation of MMP12 mRNA expression. A role of TYK2 in IL-4 signaling was also determined by Murata et al. investigating the binding characteristics of IL-4 in human colon carcinoma cell lines (34). They observed that IL-4 induces the phosphorylation of JAK1 and JAK2. TYK2 was constitutively phosphorylated. This phosphorylation was further augmented by IL-4 stimulation (34).

**Figure 7:**
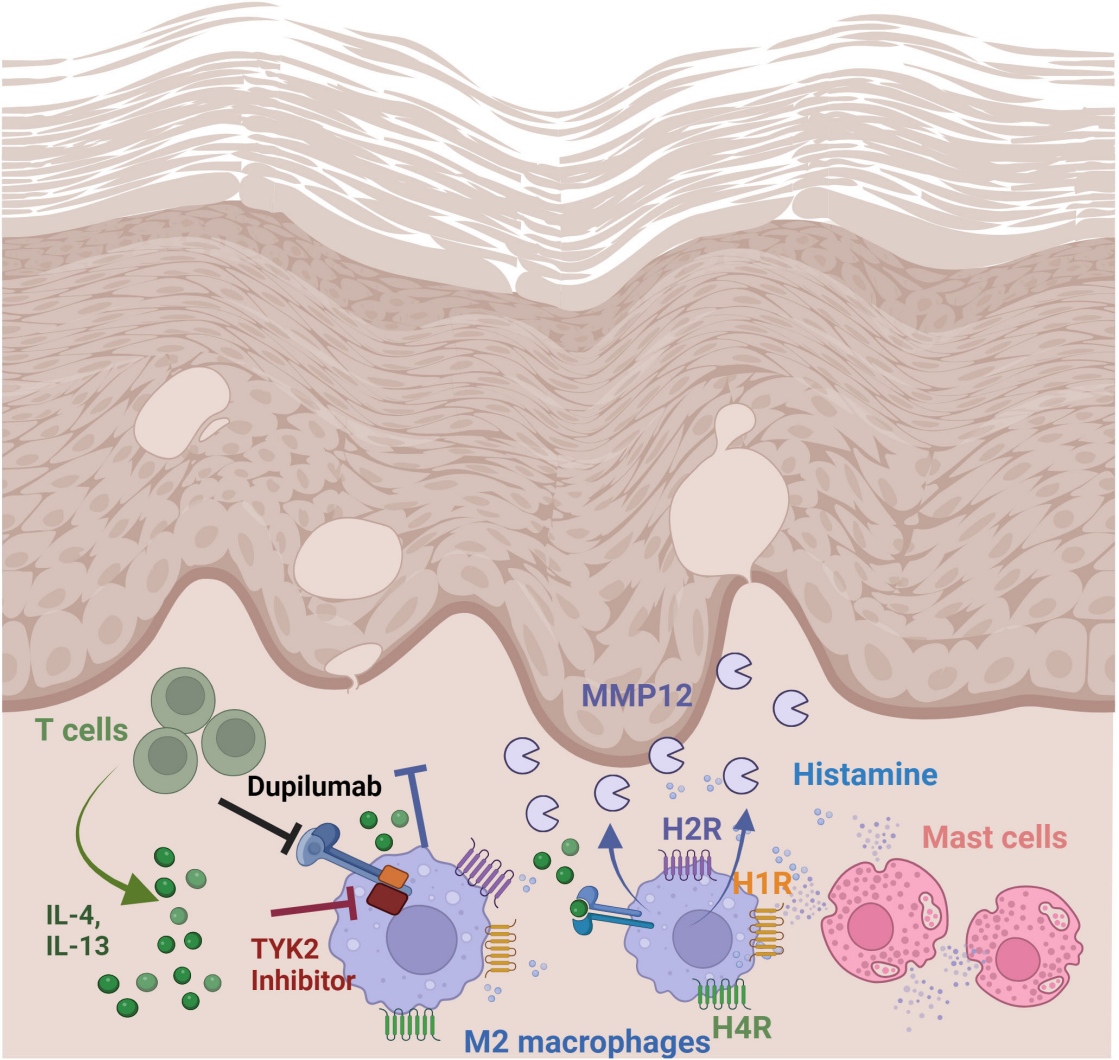
Regulation and function of MMP12 in lesional skin of AD. Summary of findings: Th2 cytokines via IL-4/IL-13 receptor type II and histamine, targeting H2R and H4R mediate a strong upregulation of MMP12 expression in M2 macrophages which is increased in lesional skin in AD. Thereby the matrix degrading enzyme MMP12 can cleave several extracellular matrix proteins which contribute to inflammatory tissue destruction. Treatment with dupilumab attenuates Th2 cytokine-induced MMP12 upregulation. Schematic overview created with BioRender.com.

Junttila stated that JAK associated with the subunits of type I or type II receptors common IL-2R gamma chain (JAK3), IL-4Rα (JAK1) and IL-13Rα (TYK2/JAK2) can auto- or cross-phosphorylate each other leading to their activation which also underscores a role of TYK2 not only in IL-13 but also in IL-4 signaling pathways via type II receptor (33). Inhibition of JAK3 and more pronounced of TYK2 prevented the upregulation of the H4R mRNA expression induced by IL-4 in human M2 macrophages (21). Differential regulation of critical inflammatory genes including 15-lipoxygenase, monoamine oxidase A and the scavenger receptor CD36 by IL-4 using IL-4Rα/JAK1/STAT3/STAT6 cascade or IL-13 using both IL-4Rα/JAK2/STAT3 and IL-13Rα1/TYK2/STAT1/STAT6 signaling pathways was observed in monocytes and alternatively activated macrophages (35). Together these data suggest that gene transcription in response to Th2 cytokines is regulated via different target selective IL-4 or IL-13 downstream signaling molecules in human macrophages. To sum up, we demonstrated a novel mechanism for mediators which are abundant in AD skin, such as Th2 cytokines via IL-4/IL-13 type II receptor and histamine targeting H4R and most pronounced the H2R. Th2 cytokines and histamine, alone or in combination, mediate a strong upregulation of MMP12 expression in M-CSF differentiated macrophages (**Figure 7**).

Importantly, the inflammatory protein MMP12 was identified as a signature protein in AD. The increased blood levels of MMP12 correlate with its expression in lesional- and non-lesional skin (36).

We speculate that the large number of M2 macrophages identified by CD68 and mannose receptor expression in inflamed AD skin (10, 37) and their interactions with Th2 cytokines and histamine are responsible for the increased levels of MMP12 at this location.

Combined therapeutic application of dupilumab and histamine receptor antagonists should be an option to disrupt this process.

## Data Availability

The raw data supporting the conclusions of this article will be made available by the authors, without undue reservation.

## Supplementary material

Supplementary Figure 1 Histamine and amthamine treatment during macrophage differentiation specifically enhance MMP12 mRNA expression without affecting the differentiation of the cells. Human monocytes were isolated from PBMCs obtained from anonymous healthy donors and differentiated into M2 macrophages in the presence of M-CSF (10 ng/ml) over 10 days. The cells were treated for 10 days with histamine and the H2R agonist amthamine while a separate group of cells remained untreated throughout differentiation. **(A)** MMP12 mRNA expression, **(B)** CD68 mRNA expression, **(C)** CD163 mRNA expression, **(D)** CCL17 mRNA expression, **(E)** CCL18 mRNA expression. mRNA expression levels were quantified by qPCR using Quantitect® primer assays from Qiagen (Hilden,Germany) and calculated by the [delta] Ct method and shown as target/reference ratios. Data are shown as individual values; horizontal bars indicate the medians. Significant differences, as determined by the Friedman Dunn’s multiple comparison test are indicated as follows: * p < 0.05; ** p < 0.01; *** p < 0.001; **(A–E)**, n = 10 independent donors and experiments); NS = non-stimulated, ns = not significant.

Supplementary Figure 2 M2 macrophages from AD patients show a significantly higher expression of MMP12 at baseline and a stronger upregulation of MMP12 mRNA expression when stimulated with IL-4 or IL-13 compared to cells from healthy donors. Human monocytes were isolated from PBMCs obtained from AD patients or from anonymous healthy donors and differentiated over 10 days into M2 macrophages in the presence of M-CSF (10 ng/ml). The cells were stimulated for 48 h either with IL-4 (20 ng/ml) or IL-13 (15 ng/ml) while a separate group of cells remained non-stimulated (NS). The data shown here for AD patients were taken from **Figure 4B** and have been compared with the data from healthy donors in **Figures 6A, B** (presented here as target/reference ratio). Target/reference ratios of MMP12 mRNA/rps 20 mRNA expression were analyzed by qPCR and calculated by the [delta] Ct method. Significant differences, as determined by the Mann-Whitney test (bars and stars in black) for NS = non-stimulated, IL-4- and IL-13-activated cells from AD patients compared to respective cells from healthy donors or by the Wilcoxon matched-pairs signed-rank test (bars and stars in orange or pink) for non-stimulated cells and activated cells with IL-4 or IL-13 in the same group are indicated as follows: * p < 0.05; ** p < 0.01; *** p < 0.001; (AD patients n = 12; healthy controls n = 14),
