# Supplementary figures and images for: Histamine and Th2 cytokines independently and synergistically upregulate MMP12 expression in human M2 macrophages
Source: Front Immunol. 2024 Oct 21;15:1429009. doi: 10.3389/fimmu.2024.1429009 (PMC11536267; doi:10.3389/fimmu.2024.1429009)

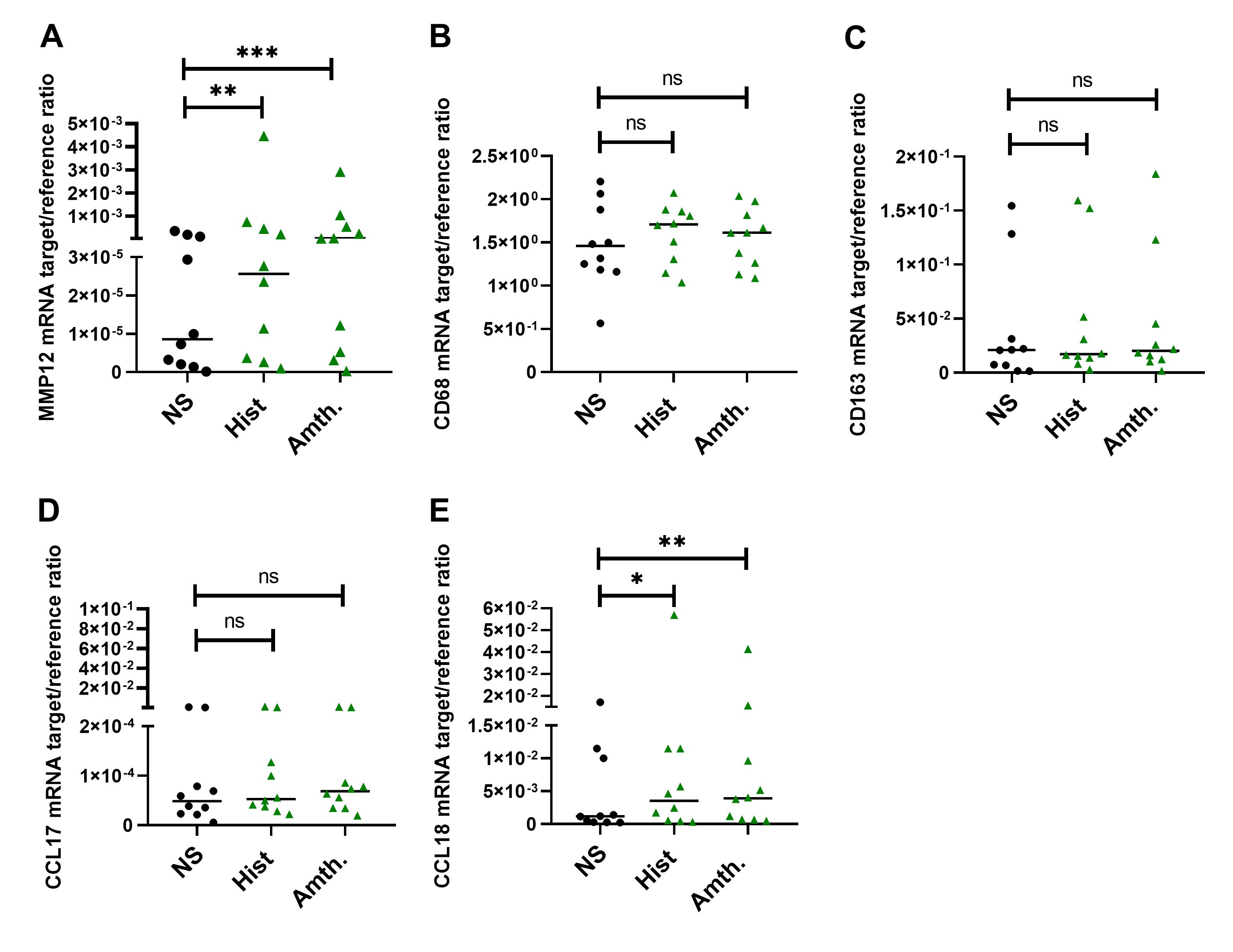

Supplement: Supplementary Figure 1 — Histamine and amthamine treatment during macrophage differentiation specifically enhance MMP12 mRNA expression without affecting the differentiation of the cells. Human monocytes were isolated from PBMCs obtained from anonymous healthy donors and differentiated into M2 macrophages in the presence of M-CSF (10 ng/ml) over 10 days. The cells were treated for 10 days with histamine and the H2R agonist amthamine while a separate group of cells remained untreated throughout differentiation. (A) MMP12 mRNA expression, (B) CD68 mRNA expression, (C) CD163 mRNA expression, (D) CCL17 mRNA expression, (E) CCL18 mRNA expression. mRNA expression levels were quantified by qPCR using Quantitect® primer assays from Qiagen (Hilden,Germany) and calculated by the [delta] Ct method and shown as target/reference ratios. Data are shown as individual values; horizontal bars indicate the medians. Significant differences, as determined by the Friedman Dunn’s multiple comparison test are indicated as follows: * p < 0.05; ** p < 0.01; *** p < 0.001; (A–E), n = 10 independent donors and experiments); NS = non-stimulated, ns = not significant. [file Image1.jpeg]

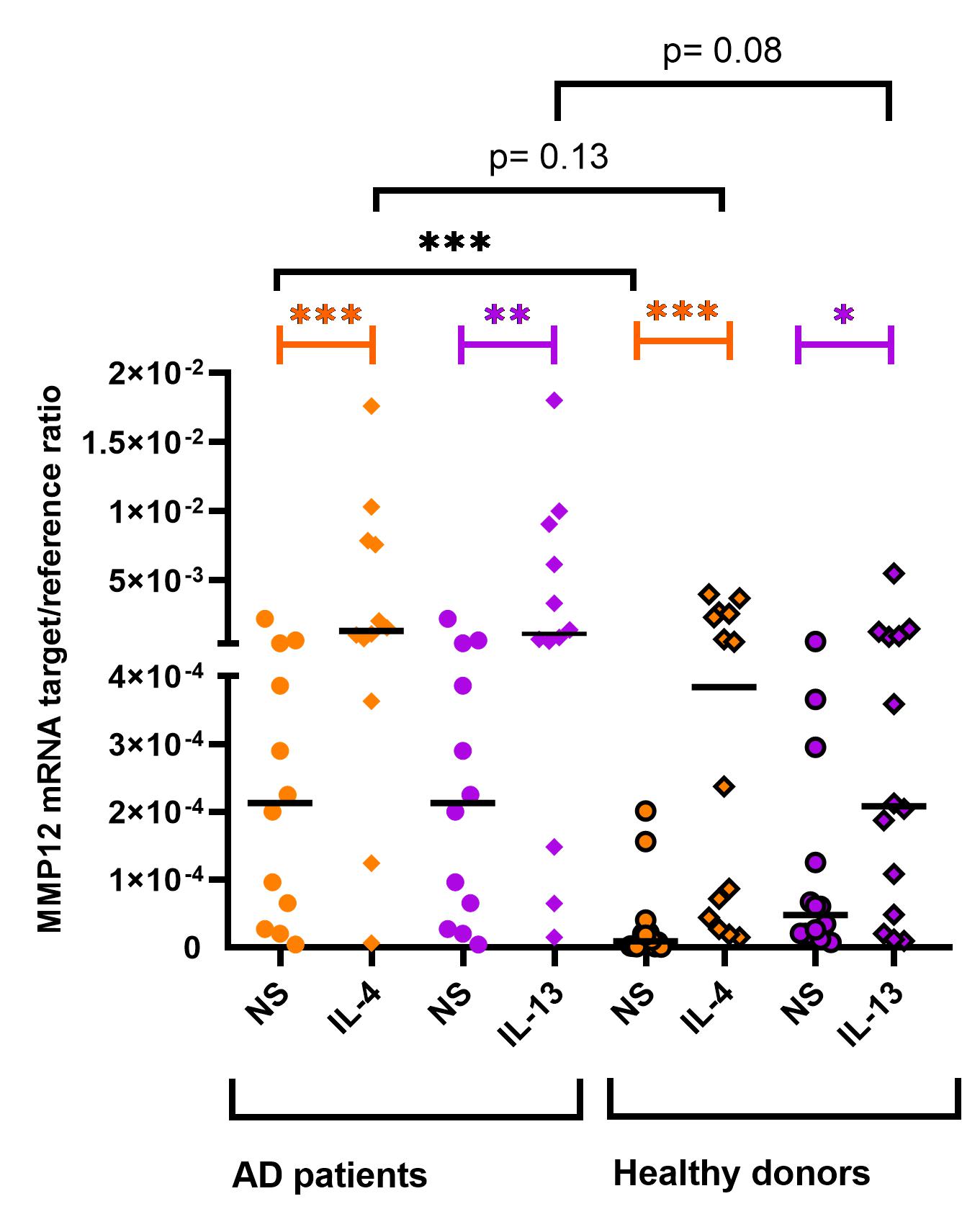

Supplement: Supplementary Figure 2 — M2 macrophages from AD patients show a significantly higher expression of MMP12 at baseline and a stronger upregulation of MMP12 mRNA expression when stimulated with IL-4 or IL-13 compared to cells from healthy donors. Human monocytes were isolated from PBMCs obtained from AD patients or from anonymous healthy donors and differentiated over 10 days into M2 macrophages in the presence of M-CSF (10 ng/ml). The cells were stimulated for 48 h either with IL-4 (20 ng/ml) or IL-13 (15 ng/ml) while a separate group of cells remained non-stimulated (NS). The data shown here for AD patients were taken from Figure 4B and have been compared with the data from healthy donors in Figures 6A, B (presented here as target/reference ratio). Target/reference ratios of MMP12 mRNA/rps 20 mRNA expression were analyzed by qPCR and calculated by the [delta] Ct method. Significant differences, as determined by the Mann-Whitney test (bars and stars in black) for NS = non-stimulated, IL-4- and IL-13-activated cells from AD patients compared to respective cells from healthy donors or by the Wilcoxon matched-pairs signed-rank test (bars and stars in orange or pink) for non-stimulated cells and activated cells with IL-4 or IL-13 in the same group are indicated as follows: * p < 0.05; ** p < 0.01; *** p < 0.001; (AD patients n = 12; healthy controls n = 14), [file Image2.jpeg]
